# The Pros and Cons of Using Algal Polysaccharides as Prebiotics

**DOI:** 10.3389/fnut.2020.00163

**Published:** 2020-09-22

**Authors:** Martin Gotteland, Karla Riveros, Naschla Gasaly, Constanza Carcamo, Fabien Magne, Gianella Liabeuf, Alejandra Beattie, Sebastián Rosenfeld

**Affiliations:** ^1^Department of Nutrition, Faculty of Medicine, University of Chile, Santiago, Chile; ^2^Department of Human Nutrition, Institute of Nutrition and Food Technology (INTA), University of Chile, Santiago, Chile; ^3^Millennium Nucleus in the Biology of Intestinal Microbiota, Santiago, Chile; ^4^Microbiology and Mycology Program, Institute of Biomedical Sciences (ICBM), Faculty of Medicine, University of Chile, Santiago, Chile; ^5^Laboratorio de Ecosistemas Marinos Antárticos y Subantárticos, Universidad de Magallanes, Punta Arenas, Chile; ^6^Centro de Investigación para la Conservación de Ecosistemas Australes, Punta Arenas, Chile; ^7^Laboratorio de Ecología Molecular, Departamento de Ciencias Ecológicas, Facultad de Ciencias, Universidad de Chile, Santiago, Chile; ^8^Instituto de Ecología y Biodiversidad, Santiago, Chile

**Keywords:** gut microbiota, laminaran, fucoidan, ulvan, carrageenan, agaran, alginate, sulfated polysaccharides

## Abstract

Macroalgae stand out for their high content of dietary fiber (30–75%) that include soluble, sulfated (fucoidan, agaran, carrageenan, and ulvan) and non-sulfated (laminaran and alginate) polysaccharides. Many studies indicate that these compounds exert varied biological activities and health-promoting effects and for this reason, there is a growing interest for using them in food products. The aim of this review was to critically evaluate prebiotic properties of algal polysaccharides, i.e., their ability to exert biological activities by modulating the composition and/or diversity of gut microbiota (GM). Pre-clinical studies show that the non-sulfated alginate and laminaran are well-fermented by GM, promoting the formation of short chain fatty acids (SCFAs) including butyrate, and preventing that of harmful putrefactive compounds (NH_3_, phenol, *p*-cresol, indole and H_2_S). Alginate increases *Bacteroides, Bifidobacterium*, and *Lactobacillus* species while laminaran mostly stimulates *Bacteroides* sp. Results with sulfated polysaccharides are more questionable. Agarans are poorly fermentable but agarose-oligosaccharides exhibit an interesting prebiotic potential, increasing butyrate-producing bacteria and SCFAs. Though carrageenan-oligosaccharides are also fermented, their use is currently limited due to safety concerns. Regarding fucoidan, only one study reports SCFAs production, suggesting that it is poorly fermented. Its effect on GM does not indicate a clear pattern, making difficult to conclude whether it is beneficial or not. Notably, fucoidan impact on H_2_S production has not been evaluated, though some studies report it increases sulfate-reducing bacteria. Ulvan is badly fermented by GM and some studies show that part of its sulfate is dissimilated to H_2_S, which could affect colonic mitochondrial function. Accordingly, these results support the use of laminaran, alginate and agaro-oligosaccharides as prebiotics while more studies are necessary regarding that of fucoidan, carrageenan and ulvan. However, the realization of clinical trials is necessary to confirm such prebiotic properties in humans.

## Introduction

Macroalgae are a large group of aquatic plants that appeared on Earth 1.6 to 1.7 billion years ago and are distributed in different climatic zones around the world (1). More than 10,000 different species of marine macroalgae have been described, which are taxonomically classified into three main Phyla: Chlorophyta (green algae), Ochrophyta (brown algae) and Rhodophyta (red algae), according to the presence of certain pigments (Table 1) (2). They are of great ecological importance due to their ability to supply oxygen to the sea, contribution to carbon cycling, and role in the marine trophic chain. From a nutritional point of view, macroalgae are low in fat (<4% of dry weight) and represent an interesting source of protein (between 15 and 25% according to the type of alga) of good nutritional value, although some species lack essential amino acids (3, 4). In addition to their high levels of carotenoids, polyphenols, vitamins, and minerals, algae are especially an excellent source of dietary fiber, i.e., non-digestible polysaccharides, that represents between 30 and 75% of their dry weight. Due to their interesting nutritional value, algae have been part of the human diet since thousands of years. Archaeological evidence suggests, for example, that they were already consumed in Chile 14,000 years BP (5). Their world production is currently over 20 million tons per year, mainly from China and Indonesia (3). Seaweed has an important culinary and medicinal use in Asia whereas in Western countries, it was until now exclusively intended for the extraction of phycocolloids widely used by the food, pharmaceutical and cosmetic industries for their rheological properties (6). However, the growing knowledge of their health benefits has spurred their inclusion as functional ingredients in a wide range of food products worldwide (3).

**Table 1:** Classification of macroalgae and presence of polysaccharides.

**Phylum**	**Genus species**	**Pigments**	**Soluble non-digestible polysaccharides**	**Polysaccharide functions**	**% Dry weight**
Ochrophyta (Brown algae)	*Macrocystis pyrifera* *Lessonia nigrescens* *Durvillae antartica* *Laminatia japonica* *Undaria pinnafitida* *Ascophyllum nodosum*	Xanthophylls (fucoxanthin, flavoxanthin)	Alginate Laminaran Fucoidan	Structural Storage Structural	30–40% 35% 5–10%
Rhodophyta (Red algae)	*Gracilaria* spp. *Porphyra* spp. *Chondrus crispus* *Mastocarpus stellatus* *Eucheuma* spp.	Phycobiloprotein (Phycoerythrin Phycocyanin. Allophycocyanin) Chlorophyll a and d	Agar Carrageenan	Structural Structural	40–50%
Chlorophyta (Green algae)	*Ulva lactuca* *Entomorpha* spp.	Chlorophyll a and b Xanthophylls (lutein, violaxanthin, neoxanthin)	Ulvan	Structural	8–30%

More particularly soluble algal polysaccharides (PS) are emerging as interesting prebiotic candidates. However, their complex chemical structure and the high presence of sulfate residues in some of them could limit their fermentation by the gut microbiota (GM) and eventually stimulate the formation of potentially harmful compounds susceptible to affect colonic function. These aspects are generally not considered in the studies analyzing the effects of algal PS. Accordingly, this review describes the different PS of marine macroalgae and examines, in a critical way, the current evidence supporting their use as prebiotics capable of exerting health-promoting effects through the GM modulation.

## Algal Polysaccharides

Polysaccharides, the most abundant components of macroalgae, are mainly involved in energy storage and structural functions. Storage PS include “real” starch in green algae, floridian starch (which exhibits an amylopectin-like structure and does not contain amylose) in red algae, and laminaran in brown algae (7). Structural PS present in algal cell wall consist in water-insoluble, high molecular weight compounds (mainly cellulose, xylans, and mannans) and water-soluble PS such as agar, carrageenan, alginate, fucoidan and ulvan, which represent the vast majority of the dietary fiber present in algae. These polymeric structures are formed by repeating units of neutral and acidic sugars linked by specific glycosidic bonds (7). Remarkably, the glycosidic linkages, monosaccharide composition, molecular weight and sulfate content can differ for the same PS, depending on the algal species and harvesting time. Unlike terrestrial plants that contain PS with the same sugars, many algae PS are sulfated (fucoidan, agar, carrageenan, ulvan), which contributes to their structural diversity and gives them specific properties. The reason why seaweed PSs are sulfated is unclear. Though sulfated PS are absent in glycophytes (salt-intolerant) plants, they are present in halophytes plants growing in high salinity soils. The concentration of sulfated PS and their degree of sulfation correlate with the concentrations of salt in the ecosystem, suggesting that sulfated PS reflect a convergent adaptation of algae and halophytes to high-salt environments (8). It has been proposed that sulfated PS work as polyanions generating higher Donnan potential by increasing ion density in the vicinity of the plant cell wall and facilitating ion transport at high salt concentrations (8). Sulfated PS might also prevent desiccation and osmotic stress at low tide, by scavenging water and ion in the extracellular matrix. On the other hand, the exposition of the green microalga *Chlamydomonas reinhardtii* to sodium nitrate enhances the concentrations and sulfation degree of its PS. This phenomenon promotes the anti-microbial and antioxidant properties of the PS, improving algal protection (9). Finally, sulfated PS also sequester heavy metals, being alginate more efficient than carrageenan and agar (10). It is unclear whether this activity facilitates metal absorption from the saline environment or reduces the deleterious effects of the heavy metals in the algal cells. These reasons probably explain why fucoidans are also found in some marine animal species such as Holothuroidea (sea cucumber), a class of echinoderm (11).

PS therefore represent an important component of macroalgae, characterized by complex structures frequently containing sulfate residues and which, overall, reflect the adaptation of these organisms to the marine environment.

## Degradation of Dietary PS in the Colon

Contrarily to starch and floridian starch which are digested by the pancreatic amylase and the enterocyte brush border disaccharidases, no enzymes in the human small intestine can hydrolyze the glycosidic bonds existing in algal PS (12). Those are therefore undigestible and reach the colon where they are metabolized by the resident microbiota. In most of the human populations in the world, the gut microbiota (GM) is dominated by two bacterial phyla, Firmicutes and Bacteroidetes, while the other phyla, mainly Proteobacteria, Actinobacteria, and Verrucomicrobia, are sub-dominant (13). GM exhibits a high inter and intraindividual variability and its microbial diversity and composition are associated with the healthy status of the host. The gut microbiome displays a wide repertoire of genes encoding for the catabolic machinery responsible for the binding and subsequent degradation of complex PS, including those from seaweeds (14, 15). These genes are clustered in Polysaccharide Utilization Loci (PUL) present in bacterial genomes. PUL include genes encoding for Carbohydrates Active Enzymes (CAZyme) belonging to the Glycoside Hydrolase (GH), Polysaccharide Lyase (PL) and Glycosyl Transferases (GT) families, and other enzymes of interest such as sulfatases (16, 17). GHs and PLs cleave glycosidic bonds in the PS through the insertion of a water molecule or by elimination mechanism, respectively. CAZyme characterization is a highly active field of investigation and currently, 167 families of GHs and 40 families of PLs are reported in the Carbohydrate-Active enZYmes Database (www.cazy.org) (18). The main CAZymes families implicated in the degradation of algal PS are described in Table 2. While these enzymes are mostly expressed in marine bacteria, some of them are also present in bacteria from the GM (19–22). Noteworthily, recent observations reported that genes encoding for porphyranases and agarases involved in the hydrolysis of porphyran and agarose respectively, have been transferred from the algal symbiont *Zobellia galactanivorans* to the human symbiont *Bacteroides plebeius* in the GM of Japanese individuals who traditionally consumed uncooked seaweed (23, 24). Consequently, these individuals have an improved colonic metabolism of these PS, this event being an interesting example of food adaptation involving GM in humans. Generalizing this observation, it is probable the CAZyme repertoire involved in algal PS degradation is larger in the GM from Asiatic populations than in that from European and North American populations. Several studies have identified PULs in the genome of human gut bacteria including *Streptococcus, Eubacterium, Bifidobacterium, Faecalibacterium* and, mostly, *Bacteroides*. *Bacteroides thetaiotaomicron* is considered as the “primary” degrader of PS since it expresses around 275 different GHs and PLs (25). These enzymes reflect the energy harvesting capacity of bacteria and their ability to adapt to a wide range of PS from endogenous (mucin) and exogenous (diet) origin. PS degradation by *B. thetaiotaomicron* generates oligosaccharides (OS) which are then used by other bacterial populations through cross feeding mechanisms.

**Table 2:** Characteristics of the algal polysaccharides and CAZymes involved in their hydrolysis.

**Soluble polysaccharides**	**Gelling properties**	**Sugars**	**Structure**	Hydrolytic enzymes^*^
Alginate MW: 30-600kDa	Yes	D-Mannuronic acid L-Guluronic acid	Homopolymeric blocks of (1–4)-linked β-D-mannuronate and its C-5 epimer α-L-guluronate residues.	PL6, PL15, PL17, PL18, PL31, PL32, PL34, PL36, PL39
Laminaran MW:4-5kDa	No	Glucose mannitol	A type of β-glucan: linear backbone of 20–30 residues of β-(1–3)-linked-D- glucopyranose with some random β-(1–6)-D- glucopyranose side chains.	GH16, GH157
Fucoidan MW: 100-1600kDa	No	Fucose, D-Xylose D-Galactose, D-Mannose Glucuronic acid Sulfate (15–30%)	Type I: α (1 → 3)- linked α-L-fucopyranose Type II: alternate α (1 → 3)- and α (1 → 4)- linked α-L-fucopyranose. Fucose-linked sulfate groups in C2- and C4- positions (Type I) or in C2-, C3- and C4 (Type II).	GH29, GH95
Agar MW: 180kDa Carrageenan MW: 200-800kDa	Yes Yes	D-Galactose Anhydrous L-Galactose Sulfate (0.5-8%) D-Galactose Anhydrous L-Galactose Sulfate (20-40%)	Sulfated galactans backbone of alternating 3-linked β -D-galactose and 4-linked a-D-galactose residues β-D-galactopyranose repetitive unit and α-D-galactopyranose.	GH2, GH16, GH86, GH117GH2, GH117, GH150, GH167GH16, GH86
Ulvan MW: 190-8200kDa	Weak	Sulfated L-rhamnose D-glucuronic acid L-iduronic acid D-xylose, Glucose Galactose, Uronic acid Sulfate (2-40%)	Repeating units of ulvanobiouronic acid Types A (α-L-rhamnose 3-sulfate-1, 4- β-D-glucuronic acid), ulvanobiouronic acid Type B (α-L-rhamnose 3-sulfate-1, 4-α-L-iduronic acid), and α-L-rhamnose-3-sulfate-1, 4β-D-xylose.	GH78, GH145, PL24, PL25, PL28, PL40

* *Modified from Cherry et al. (4)*.

*MW, molecular weight; GH, glycoside hydrolase; PL, polysaccharide lyase*.

The human GM can be therefore considered as a reservoir of enzymes allowing, among other, the degradation of non-digestible PS including those of algae, and whose diversity varies from one population to another.

## The Concept of “Prebiotics”

The concept of prebiotic refers to food ingredients that are not hydrolyzed by the pancreatic and intestinal enzymes and reach the colon where they are fermented by the resident microbiota, stimulating selectively the multiplication and/or activity of one or several health-promoting bacterial populations (26). Prebiotic compounds are mostly non-digestible, soluble, carbohydrates, but recent studies suggest that dietary phytochemicals could also display prebiotic properties (27, 28). It must be stated that not all soluble fibers are prebiotics. In fact, a soluble fiber is not a prebiotic when it is not or badly fermented by the GM and remains practically non-metabolized in the colon. This is the case for some complex PS including some algae PS, as we will see later in this review. A great diversity of prebiotic, natural or synthetic, are currently available in the world market, being fructans [inulin and fructo-oligosaccharides (FOS)] the most studied and used in foods (29–31). The prebiotic effect is exclusively mediated by the regulatory activities of the dietary compound on the microbiota and the subsequent positive impact on host's health. This may occur by stimulating the growth of health-promoting bacterial populations including *Lactobacillus* spp., *Bifidobacterium* spp., *Akkermansia muciniphila*, or butyrate-producing bacteria, and the subsequent formation of beneficial metabolites, mainly short chain fatty acids (SCFAs). On the other hand, this may also occur by inhibiting the growth of pathogens or pathobionts in the colon, and/or by reducing the formation of metabolites such as NH_3_, phenols, p-cresol, skatole, hydrogen sulfide (H_2_S) (32, 33), or trimetyl-amine that are potentially harmful for the colonic mucosa and, when absorbed, for the kidneys and the vascular endothelium. Regarding SCFAs, they exert a great array of physiological effects, not only in the colon but also in different tissues and organs including the immune system, through the stimulation of specific G protein-coupled receptors: Gpr41, Gpr43, and Gpr109a, present in these different tissues (34). Thereby prebiotics, through the formation SCFAs and the stimulation of these receptors, contribute to the regulation of intestinal motility and bowel habits, and stabilize the gut barrier function by increasing the expression of tight-junction proteins, thus contributing to the homeostasis of the digestive ecosystem (35). They also attenuate metabolic alterations (glucose intolerance, insulin resistance, metabolic endotoxemia, dyslipidemia, hypertension and low-grade inflammation), increase the secretion of anorexigenic hormones and incretins, stimulate neurochemical-producing bacteria and the gut-brain axis, and improve calcium absorption and bone health. Finally, prebiotic-derived SCFAs also stimulate the immune system and exert anti-inflammatory activities by inhibiting NFKB pathway, reinforcing the defense of the individuals and reducing the development of allergic and inflammatory diseases (36). The mechanisms of action of prebiotics at molecular and cellular level were the subject of several recent reviews (37, 38). However, it is important to state that soluble dietary fiber can also exert physiological effects through mechanisms independent of their prebiotic effects.

The prebiotic effect of a given PS therefore depends on its ability to stimulate beneficial bacteria exerting directly or indirectly, through the production of SCFA, beneficial effects for health, or to reduce deleterious bacteria capable of producing potentially toxic metabolites.

## The Evaluation of PS Prebiotic Activity

The prebiotic potential of a PS can be evaluated through different preclinical models. Culture of pure bacterial strains with the PS as sole source of carbon is frequently used but it only allows the detection of bacteria acting as primary degrader, not these indirectly implicated in its degradation through cross-feeding interactions. More complex systems use bacterial consortia directly or indirectly involved in the PS degradation (39). This approach includes the use of genomic-scale metabolic models (GSMs) and algorithms for microbial community design. It allows to quickly obtain theoretical compositions of consortia capable of degrading the PS and produce metabolite(s) of interest. Many studies used bioreactors in which animal or human fecal microbiota are cultured with the PS in controlled conditions (33). More sophisticated and integrated systems allow the anaerobic culture of complex microbiota in presence of epithelial cell monolayers, mimicking therefore in a more real way the colonic ecosystem (40). On the other hand, the use of animal, mice, rats, or pigs, allows to determine simultaneously the changes induced by the PS on the GM composition, bacterial metabolite production, and host physiological parameters. Animals can be fed normal or high-fat, high sucrose, or high protein diets to mimic metabolic alterations like these observed in humans. Finally, germ-free animals colonized with human fecal microbiota are also used to explore the physiologic impact on the host. The results observed in animals are, however, difficult to extrapolate to humans and clinical trials are needed to confirm the prebiotic activity of the PS. Intriguingly, while many studies using prebiotic PS from terrestrial plants have been conducted in human volunteers, human studies using algae PS are scarce.

Regarding the methods used to characterize GM composition, it is important to consider that most of the microbial taxa are uncultivable, limiting the use of the classical culture methods. Accordingly, nucleic acid-based approaches [fluorescent *in situ* hybridization (FISH), qPCR, or temperature gradient gel electrophoresis (TGGE)] have been developed to circumvent the cultivation step. However, they are frequently restrictive as focused on specific bacterial populations.

In consequence, Next Generation Sequencing, consisting in the identification of genomic DNA or phylogenetic markers (16S rDNA), provides a more global vision of the GM composition and allows the detection of low-abundance bacterial taxa, being currently considered as the gold-standard method (41).

## Some Concerns About the Use of Algal PS as Prebiotics

Before to describe the different studies exploring the prebiotic effect of algal PS, it must be stated that their use in humans could be limited by two recently emerged concerns related to their sulfate content and thickening/emulsifying properties.

### Sulfate Content and H_2_S Production

The colonic supply of free sulfate depends on dietary intake through water and foods, and the *in situ* microbial degradation of sulfated compounds including sulfomucins, heparan sulfate, or chondroitin sulfate (42, 43). Fucoidan, agar, carrageenan and ulvan are highly sulfated PS and their eventual degradation by the microbiota might contribute to increase the levels of free sulfate in the colon. Some *Bacteroides* species express exo- and endo-sulfatases capable of desulfating simple and complex carbohydrates (44). The released sulfate may be cross-fed by sulfate-reducing bacteria (SRB) such as *Desulfovibrio*, a member of the Proteobacteria phylum, conducing to the formation of hydrogen sulfide (H_2_S) (42, 43). H_2_S is the sole inorganic substrate used by the colonocyte mitochondria (32, 45). In low concentrations, H_2_S is detoxified by the mitochondrial sulfide oxidizing unit while in high concentrations, it inhibits the mitochondrial complex IV, reducing butyrate oxidation and oxygen consumption. This favors the diffusion of oxygen to the lumen, lowering the local anaerobiosis and contributing to the overgrowth of facultative anaerobic Enterobacteria, known to be involved in the development of mucosal inflammatory processes. Interestingly, sulfatase genes from *B. thetaiotaomicron* are essential to trigger colonic inflammation in genetically susceptible mouse model (46) and increased sulfatase activity and H_2_S concentrations have been detected in fecal samples from patients with ulcerative colitis, suggesting that this compound is involved in the initiation and/or maintenance of this disease (43, 47).

Globally, these findings suggest that it is probably important to control the abundance of sulfatase-expressing bacteria, sulfate-reducing bacteria as well as the levels of H_2_S in studies carried out with highly sulfated PS. Notably, some prebiotic compounds have been shown to reduce H_2_S production by the GM (48).

### Thickening/emulsifying Properties of PS and Mucus Disruption

Alginate, agar and carrageenan display gelling properties allowing their use as thickeners and emulsifiers in food processing. Synthetic emulsifiers have been shown to increase bacterial translocation across epithelial monolayers *in vitro* (49) and Chassaing et al. (50) recently described that the administration of carboxymethyl cellulose and polysorbate 80 in rodents promoted microbiota encroachment on the mucus layer, bacterial translocation, and changes in GM composition and functionality. More particularly, an increase in the metabolic pathways involved in the synthesis of flagellin and LPS associated with the development of low-grade inflammation and metabolic syndrome was reported in these animals.

It is currently unknown whether natural thickeners including algal PS can exert similar effects. Studies are therefore needed to confirm their safety regarding this specific point.

## The Algal PS as Prebiotic Candidates

Seaweeds are a rich source of complex PS that have wide technological uses and can be considered as new prebiotic candidates (51, 52). Health-promoting activities have been reported for many of them, beyond their eventual prebiotic activities. Based on these results, fucoidans have been accepted as “Novel foods” by the European Food Safety Authorities (EFSA). Several approaches can be used for their extraction/purification. The most frequently used involve chemical hydrolysis and alkaline extraction, organic solvents, physical methods such as ultrasounds, and the use of specific enzymes (53). The latter, in addition to eliminate contaminant proteins of polyphenols frequently bound to algal PS, can also generate medium- or low-molecular weight OS, more easily fermentable by the GM than the native PS (54).

Next, we will revise the studies evaluating the prebiotic activity of the different types of algal soluble dietary fibers. With this aim, an extensive search of the literature was carried out in the Pubmed-Medline, Web of Science, and EMBASE databases, using the following search terms: “seaweed polysaccharide,” “algae polysaccharide,” “laminaran” “fucoidan,” “carrageenan,” “agaran,” “ulvan,” “alginate,” “gut microbiota,” “prebiotic,” eventually with the Boolean operators “AND” and “OR.” The search was performed on February-March 2020. The following selection criteria were applied: original publications in English language; *in vitro*, animal, or human studies evaluating the effect of algal PS on specific bacteria in culture, or animal or human microbiota. Only studies carried out with purified algal PS or algal extract enriched with a determined PS were selected. Reviews and editorials were excluded from the search as well as studies using mixtures of different PS or whole dried algae, which contain other compounds that could affect the microbiota. Studies evaluating the health effect of algal PS without studying their impact on microbiota were not considered as they do not allow to conclude about the PS prebiotic property. After selection, 40 publications were included in this review.

### Non-sulfated PS

#### Alginate

Alginates are linear PS formed by β-D-mannuronic acid and α-L-guluronic acid units with a molecular weight (MW) between 30 and 600 kDa (Table 2). They are widely used as thickeners, stabilizers, and emulsifiers in food elaboration.

Using different species of *Bacteroides* isolated from human GM, alginate was shown to be fermented only by *B. ovatus* (55). In anaerobic culture in bioreactor with human fecal microbiota for 72 h, alginate from *Laminaria japonica* increased the concentrations of acetate and propionate and stimulate the growth of *Bacteroides finegoldii* (56). Since alginate is also produced by bacteria such as *P. aeruginosa*, the *in vitro* degradation of algal and bacterial alginates was compared (57). Both PS were fully fermented by strains of *Bacteroides xylanisolvens* expressing alginate lyase genes, promoting their growth. The production of SCFAs was comparable between both types of alginate, being that of propionate, butyrate and total SCFA higher than that in the starch-containing medium used as control. The ability of *B. xylanisolvens* to degrade alginate and produce SCFA (mainly acetate and propionate) was confirmed by Li et al. as well as that of *B. ovatus* and *B. thetaiotaomicron* isolated from Chinese subjects (58). The 2-week dietary supplementation with alginate (2% W/W) of rats altered their GM by increasing alginate-fermenting bacteria, more particularly *Clostridium orbiscindens* (59).

To improve the prebiotic properties of alginate, some studies used alginate-OS which are more easily fermented. Thereby, Ramnani et al. compared 3 alginate-OS of different MW (High: 212 kDa; Medium: 97 kDa; Low: 38 kDa) which were cultured for 24 h with human fecal samples (60). While no changes were detected in the studied bacterial populations, higher concentrations of total SCFAs, acetate and propionate were observed with the high MW alginate while the medium MW only increased acetate and total SCFA and the low MW alginate did not affect SCFA production. Therefore, SCFA production was inversely proportional to the OS MW. Other study evaluated the effect of a mix of alginate-derived tri-and tetrasaccharides in pure cultures of *B. bifidum* and *B. longum*, compared with 5% FOS (61). *Bifidobacterium* growth was greater with the alginate mix than with FOS, suggesting a better prebiotic effect. This alginate mix was subsequently administered to rats in different proportion (0.5, 2.5, and 5%) for 2 weeks. The best effect was observed in the group supplemented with 2.5% of alginate-OS, in which fecal bifidobacteria increased by 13-fold and 4.7-fold compared with the control animals and those supplemented with FOS respectively, confirming the *in vitro* effect. A similar effect was observed for *Lactobacillus* sp. whose abundance increased by 5-fold, while these of enterobacteriaceae and enterococci decreased.

In another study in mice, supplementation with poly-mannuronic acid (150 mg/kg per day) for 3 months prevented the diminution of propionate and butyrate induced by a high-fat diet (HFD) (62). In addition, alginate restored the Actinobacteria phylum and decreased the Bacteroidetes and Proteobacteria phyla in the GM of the animals, reducing by 290-fold the level of *E. coli*. The higher abundance of Actinobacteria was mainly due to *B. pseudolongum* whose abundance increased from 6.4 to 33.8% with alginate. *Lactobacillus* and butyrate-producing bacteria (*Roseburia, Anaerofustis, R. bromi*) were also more abundant in the alginate-supplemented animals. Interestingly, these changes in the GM were accompanied by the normalization of metabolic and inflammatory parameters including endotoxemia, glycemia and the colonic expression of TNFα and IL-10. In the second study carried out in mice fed a HFD, alginate-OS supplementation for 10 weeks favored the growth of *A. muciniphila, L. reuteri*, and *L. gasseri*, and increased the concentrations of acetate, propionate, and butyrate (63). Alginate reversed the increase of Deferribacteres, Bacteroidaceae, Ruminococcaceae, and Lachnospiraceae, and the decrease of Erysipelotrichaceae induced by HFD, contributing therefore to the normalization of the microbiota in these animals, similarly to the previous study. The supplementation also improved metabolic and inflammatory markers including endotoxemia. Remarkably, many of these changes correlated with changes in the abundances of the different bacterial taxa. These results suggest that the effect of alginate-OS on the host metabolism might be mediated through their impact on the microbiota. Taken together, these results support an interesting prebiotic potential for alginate-OS for the prevention and dietary management of metabolic diseases.

On the other hand, some studies also determined the effect of alginate on the production of harmful bacterial metabolites issued from protein fermentation. Thereby, a 48 h-culture of human fecal microbiota with 3% soy protein and alginate increased the production of propionate but decreased that of NH_3_, phenol and indole (64). In rats fed a high protein diet supplemented with alginate (2% W/W) for 2 weeks, these authors only reported a decrease in lactate and indole formation. A similar effect was described with low MW alginate (49 kDa) fermented with human fecal microbiota: the formation of acetate and propionate were stimulated while that of NH_3_, phenol and indole was inhibited (65). When administered to rats (2% W/W), the fermentation pattern of this low MW alginate agreed with that observed *in vitro*, but only a decrease of H_2_S was detected. Similarly, a higher production of total SCFAs and a concomitant reduction of cecal H_2_S, phenol, indole and NH3 were also reported in rats supplemented with alginate (2%) for 2 weeks (66). These events were accompanied by an increased abundance of *Bacteroides capillosus*.

Interestingly, one study using alginate was carried out in healthy volunteers (67). The PS (10 g/d) was administered for 2 weeks and some bacterial populations from the fecal microbiota were analyzed by culture methods. Results only showed an increase of bifidobacteria, without significant changes in the other bacterial populations studied. Alginate increased the fecal concentrations of acetate and propionate and decreased these of H_2_S, phenol, p-cresol, indole, skatole, and ammonia, confirming therefore the results from *in vitro* and animal studies.

In conclusion, preclinical studies indicate that algal alginate is fermented by the GM, promoting the growth of *Bacteroides, Bifidobacterium*, and *Lactobacillus* species and the formation of SCFAs including butyrate. In addition, alginate tends to decrease enterobacteria and attenuates the formation of potentially harmful putrefactive compounds by the microbiota. In animal models of metabolic syndrome, alginate supplementation is associated to the restoration of GM composition and metabolic improvement. At last but not least, part of these results was confirmed in a human study. Accordingly, alginate and alginate-OS could be considered as prebiotic. Results from alginate studies are summarized in Table 3.

**Table 3:** *In vitro*, animal, and human studies evaluating the effect of no-sulfated algal polysaccharides (alginate and laminaran) on the gut microbiota and health.

**Algal polysaccharides**	**Model**	**Changes in bacterial populations**	**SCFA production**	**Putrefactive metabolites**	**Health effects**	**References**
Alginate, alginate-OS, poly-mannuronic acid	***In vitro*****:** - Pure cultures - Bioreactor with human GM	↗*Bacteroides* ↗*Bifidobacterium* ↗*Lactobacillus*	↗ SCFAs including butyrate	↘phenol, indole, NH3	Not applicable	(55–58, 60, 61, 64, 65)
	**Animal** - Rats with normal diet - Mice with HFD - Rats fed HPD	↗*Butyrate-producing bacteria* ↗*Bifidobacterium* ↗*Lactobacillus* ↗*Bacteroides* ↗*A. muciniphila* ↘*Enterobacteriaceae*	↗SCFAs	↘phenol, indole, NH3, H_2_S	Normalization of metabolic and inflammatory parameters in animals fed HFD	(59, 61–64, 66)
	**Human**	↗*Bifidobacterium*	↗ Acetate, Propionate	↘H_2_S, phenol, *p*-cresol, indole, skatole, NH_3_	Not reported	(67)
Laminaran	***In vitro*****:** - Pure cultures - Bioreactor with human GM	↗*Bacteroides* ↗*Lactobacillus*	↗ SCFAs mainly butyrate and propionate,	↘H_2_S, phenol, p-cresol, indole, skatole, NH_3_	Not applicable -	(55, 64, 65, 68–70)
	**Animal** - Rats fed ND - Rats fed a HPD or HFD - Post weaned piglets	↗ Bacteroides ↗*Coprobacillus* ↗*P. distasonis* ↗*Prevotella* ↘Enterobacteriaceae ↗GH, PL and GT expressing bacteria	↗SCFAs mainly butyrate, propionate, and lactate	↘H_2_S, phenol, indole	↗colonic expression of neutral mucins ↗ileal expression of glucose transporters ↘Inflammation markers	(59, 64–66, 71–74)

*GM, gut microbiota; H_2_S, hydrogen sulfide; HFD, high fat diet; HPD, high protein diet; ND, normal diet; GH, glycoside hydrolase; PL, polysaccharide lyase; GT, glycosyl transferase; SCFA, short chain fatty acids*.

#### Laminaran

As reported above, laminaran is the only soluble, undigestible, storage PS found in algae. It is formed by a linear backbone of 20–30 residues of β-(1–3)-linked-D-glucopyranose with some random β-(1–6)-D-glucopyranose side chains (Table 2).

This ß-glucan was fermented by strains of *B. tethaitaomicron, B. distasonis, Bacteroides* 0061-1 and *Bacteroides* T4-1 isolated from human colon (55). After 24 h culture with human fecal microbiota, it was extensively degraded (about 97%, like FOS) with a concomitant pH decrease, without inducing changes in the *Bifidobacterium* and *Lactobacillus* populations (68). Notably, these authors also reported that laminaran increased the expression of neutral mucins in the rat colon, an event which has been linked to changes in CAZyme expression by the gut bacteria. The production of SCFAs, mainly propionate and butyrate, was also incremented, compared to glucose, as previously described (69). In another study, a 2-weeks administration of laminaran (2% W/W) to rats increased the abundances of *C. ramosum* and *Parabacteroides distasonis* and the formation of butyrate and propionate (66). These changes were accompanied by the decrease of indole, phenols and H_2_S in the cecum of the animals. Although laminaran was previously shown to promote the growth of *Bifidobacterium* spp. and *Peptostreptococcus* spp., no increase of these bacteria was observed in this study (70). The impact of laminaran on the reduction of putrefactive compounds was confirmed by Nakata el al. using high soy protein content in *in vitro* and animal studies (64). A higher lactate production and a lower pH were also reported in these experiments, and the cecal microbiota of the laminaran-fed animals was characterized by a higher abundance of *Coprobacillus* (~20%), a lactate-producing bacteria, and a lower abundance of *Helicobacter* and *Parabacteroides*. These authors also confirmed the suppression of indole, p-cresol and H_2_S production and the stimulation of lactate, butyrate, and propionate formation in presence of laminaran *in vitro* and in animals (65).

Several studies have evaluated the impact of laminaran administration in post-weaned piglets. The first study showed an increased ileal expression of the glucose transporters GLUT1, GLUT2, and SGLT1, resulting in a better growth performance of the animals (71). No changes were observed in the bacterial populations specifically evaluated in the study (*Lactobacillus, Bifidobacterium*, and *E. coli*), but a significant decrease of fecal propionate was detected. In the second study, laminaran (300 ppm in diet) increased cecal total SCFAs and acetate and decreased the colonic expression of the proinflammatory cytokines IL-1β, IL-6, and IL-17A, and that of the anti-inflammatory IL10. This was accompanied by a lower count of potentially pathogen strains of attaching and effacing *Escherichia coli* (AEEC) while *Lactobacillus* and Enterobacteriaceae were not affected (72). The third study used 16S rRNA gene sequencing to evaluate the microbiota after laminaran supplementation (73). Compared to control animals, the supplemented pigs had lower abundances of Enterobacteriaceae and higher abundance of *Prevotella*, with more production of acetate and butyrate. In mice fed a HFD supplemented (1%), or not, with laminaran for 1 month (74), species richness was not affected but the Firmicutes phylum decreased and the Bacteroidetes increased, more specially the genera *Bacteroides*, and *Parabacteroides*, as previously reported (59, 66). A metagenomic analysis indicated higher expression of GHs, PLs and glycosyl transferases (GTs) in the microbiome of the supplemented animals, being GH2, GT2, GT4, PL1, and PL10 the most affected families.

Based on these studies, it can be stated that laminaran is fully metabolized by the GM. This process results in the increased formation of SCFAs (mainly butyrate and propionate) and the concomitant acidification of the colonic lumen, while it attenuates the formation of potentially harmful protein-derived metabolites. Laminaran fermentation mostly involves bacterial taxa belonging to the Bacteroidetes phylum, and butyrate-producing bacteria from the Firmicutes phylum, without implications of the genera *Bifidobacterium* and *Lactobacillus*. However, no human studies have been performed to confirm the results of the pre-clinical evaluations. Results from laminaran studies are summarized in Table 3.

### Sulfated PS

#### Fucoidan

Fucoidans are L-fucose enriched, sulfated, PS which also contain few other sugars including xylulose, glucuronic acid, mannose, and galactose (Table 2). Their sulfate contain varies between 15 and 30% according the species of algae. Two main structures have been described for fucoidans: type I fucoidan (for example from *Laminaria japonica*) composed of α (1 → 3)- linked α-L-fucopyranose and type II fucoidan (f.e. from *Ascophyllum nodosum*) composed of alternate α (1 → 3)- and α (1 → 4)- linked α-L-fucopyranose (7). Fucose-linked sulfate groups are found in C2- and C4- positions in type I fucoidan and in C2-, C3-, and C4- in type II fucoidans. It has been proposed that this special chemical structure could explain their anti-diabetic, anti-obesity, anti-inflammatory, anti-coagulation, antioxidant, anti-microbial and anti-tumoral properties (75).

One of the first studies published about fucoidan degradation by human GM was carried out by Salyers et al. (55) using several strains of *Bacteroides* spp. from human stool samples. None of them could ferment this PS. In a French study using fucoidan from *Ascophyllum nodosum* cultured in bioreactor, no production of gas and SCFA and no disappearance of this PS was observed after 24 h, suggesting that fucoidan was resistant to fermentation by human GM (69). A similar experiment was carried out using low (<30 kDa) and high MW (>30 kDa) fractions of fucoidan isolated from *L. japonica* and fermented for 48 h by fecal microbiota from Chinese subjects (76). In opposition with the previous study, a strong acidification (>1 pH unit) was observed accompanied by an increased production of acetate, butyrate and lactate and higher counts of *Bifidobacterium, Lactobacillus* and *Enterobacter*. These changes were more pronounced with the low MW fucoidan fraction, indicating that it is more easily fermented that the high MW fraction. These contradictory results could be due to the origin of the stool donors, the GM of the Chinese volunteers being probably more adapted to fucoidans, due to their traditional consumption of algae, than that of the French subjects whose diet is practically free of them.

The other studies with fucoidan were carried out in animal models. Administered to post-weaned piglets for 8 d, fucoidan was shown to reduce enterobacteria and the production of ramified SCFAs (isobutyrate and isovalerate) originated from protein fermentation, indicating a reduction of this process. However, SCFA concentrations were not affected by the treatment, suggesting that fucoidan was poorly fermented by these animals (72). Similar results were reported in rats supplemented (2%) with fucoidan from *Cladosiphon okamuranus* for 2 weeks. No fucoidan-fermenting bacteria were detected and in addition, the cecal weight of these animals was increased by 3 folds, compared with controls, and half of them suffered diarrhea (66).

Type I and Type II fucoidans isolated from *L. japonica* and *Ascophyllum nodosum*, respectively FuL and FuA, were administered to mice (100 mg/kg/day by gavage) for 6 weeks (77). High throughput sequencing showed a higher abundance of *Lactobacillus* and a lower of the pathobiont *Peptococcus* with FuA, while Ruminococcaceae increased and *A. muciniphila, Alistipes*, and Clostridiales decreased with FuL. The administration of these fucoidans to mice fed a HFD did not influence the low microbial diversity observed in these animals but increased the abundance of *Bacteroides, Akkermansia muciniphila*, and *Desulfovibrio* (78). In addition, an attenuation of the metabolic (weight gain, fat mass, energy intake, total cholesterol, triglyceridemia, fasted glycemia, and insulinemia) and inflammatory (plasma LPS-Binding protein, TNF, IL1-ß, MCP-1) alterations induced by the HFD was observed. No correlations were determined between the metabolic and inflammatory parameters and changes in bacterial taxa.

In other study carried out in rats fed a HFD supplemented with fucoidan (100 mg/kg) for 8 weeks, a lower abundance of Firmicutes and Actinobacteria phyla and higher of Bacteroidetes and Proteobacteria was observed, compared with the non-supplemented HFD animals (79). At the genus level, the relative abundance of *Clostridium, Corynebacterium, Staphylococcus*, and *Lactobacillus* decreased whereas that of *Bacillus, Ruminococcus, Adlercreutzia, Prevotella, Oscillospira, Enterobacter*, and *Desulfovibrio* increased in the fucoidan group. Interestingly, fucoidan prevented the decrease of bacterial bile salt hydrolase activities induced by the HFD in the microbiota. Indeed, bile salt hydrolase-expressing bacteria are involved in the equilibrium between primary and secondary biliary acids, that is involved in metabolic regulation in the host. Accordingly, this finding could explain the improvement of blood lipids and hepatic steatosis in the fucoidan supplemented animals.

A similar study was carried out in mice fed a HFD for 8 weeks and supplemented by gavage with 50 or 100 mg/kg/d of fucoidan (80). Fucoidan amplified the reduction of alpha-diversity induced by the HFD, decreased the Firmicutes phylum and increased Proteobacteria. GM from the fucoidan-treated mice (100 mg/kg) was enriched in *Desulfovibrio, Helicobacter, Mucispirilum*, and Rumicococcaceae. Although fucoidan administration alleviated dyslipidaemia in the animals, no clear beneficial effect was observed on GM composition.

Finally, the impact of fucoidan from *Sargassum fusiforme* was evaluated on streptozotocin-treated mice, a model of type 1 diabetes, after 6 weeks of supplementation (81). The diabetic mice displayed higher fasting glycemia and food and water intake, associated with cardiac and hepatic alterations and a higher abundance of Proteobacteria and Firmicutes/Bacteroidetes ratio, a controversial marker of obesity (82). At the genus level, they also exhibited increased level of *Lactobacillus* and *Bifidobacterium* and lower microbial diversity. These metabolic and microbial alterations were partially prevented by fucoidan administration and more specifically, the relative abundance of *Alloprevotella, Alistipes, Odoribacter, Millionella, Roseburia, Erysipelatoclostridium, Aerococcus, Rikenella, Lachnoclostridium*, and *Acetatifactor* was enhanced.

In conclusion, results from the fucoidan studies are more heterogenous and contradictory. It is difficult to conclude whether this PS is fermented, or not by the microbiota as SCFA production was observed in only one study, while two studies described an absence of production and the remaining studies did not report this data. Though fucoidans are highly sulfated and that two studies using high throughput sequencing reported increased abundances of the sulfate-reducing bacteria *Desulfovibrio*, no studies evaluate the impact of this PS on the production of H_2_S. The impact on the microbiota is highly variable according the study, and no clear pattern can be demonstrated, making difficult to conclude whether it is beneficial or not. The studies using animal models of metabolic diseases show improvement with fucoidan supplementation, but it cannot be concluded that such effects are due to the regulation of the microbiota. In addition, a high frequency of adverse effects was reported in one study. Accordingly, more studies are necessary to determine whether fucoidan can be considered as prebiotic compounds and to confirm its safety regarding H_2_S production. Results from fucoidan studies are summarized in Table 4.

**Table 4:** *In vitro* and animal studies evaluating the effect of sulfated algal polysaccharides (fucoidan, agaran/carrageenan, ulvan) on the gut microbiota and health.

**Algal polysaccharides**	**Model**	**Changes in bacterial populations**	**SCFA production**	**Putrefactive metabolites**	**Health effects**	**References**
Fucoidan Low MW fucoidan Type I fucoidan Type II fucoidan	***In vitro*****:** - Pure cultures, - Bioreactor with human GM	Not fermented by *Bacteroides* sp. or human GM ↗*Bifidobacterium, Lactobacillus* and *Enterobacter*	Low or no production ↗acetate, butyrate, lactate	Not evaluated	Not applicable	(55, 69, 76)
	**Animal** - Post-weaned piglets - Rats, Mice fed HFD - Mice with type 1 diabetes	Contradictory results ↗bile salt hydrolase expressing bacteria ↗Desulfovibrio	Not affected or not reported	Not evaluated	50% of the animals with diarrhea ↘metabolic and inflammatory alterations Normalization of metabolic alterations in diabetic mice	(66, 72, 77–81)
Agaran Agaran-OS Carrageenan Carrageen	***In vitro*****:** - Pure cultures, - Bioreactor with human GM	↗ Butyrate produ-cing bacteria ↗*Desulfovibrio* ↗*Bifidobacterium* and *Lactobacillus*	↗SCFA including butyrate	Not evaluated	Not applicable	(60, 83–87)
	•Exercise-induced fatigue in mice Mice with ND Mice with colitis	↗ Butyrate produ-cing bacteria ↗*Bifidobacterium* and *Lactobacillus*	↗SCFA including butyrate	Not evaluated	Prevent alterations induced by exercise (glycogen storage, oxidative stress, plasma lactic acid and gut epithelial integrity).	(85, 88–90)
Ulvan	***In vitro*****:** - Pure cultures, - Bioreactor with human or animal GM	*Bacteroides* *Lactobacillus* *Bifidobacterium* (only 1 study)	Poor fermentation and low SCFA production	↗ H_2_S	Not applicable	(91–93)

*GM, gut microbiota; H_2_S, hydrogen sulfide; HFD, high fat diet; ND, normal diet; SCFA, short chain fatty acids*.

#### Agarans/Carrageenans

The carrageenan structure consists of a β-D-galactopyranose repetitive unit and α-D-galactopyranose (Table 2). Their degree of sulfation is higher than that of agarans and the number and position of sulfate groups determine their structure and properties (7). Fifteen types of carrageenan have been described, of which kappa (κ)-, iota (ι)- and lambda (λ) are those with the largest commercial interest. Agarans are formed mainly of alternating 3-β-D-galactopyranose and 4-(3, 6)-anhydro-α-L-galactopyranose units (7). Their structure varies according the presence of sulfate, methoxy and/or pyruvic groups. Porphyrans are a class of agaran synthesized by the *Pyropia, Porphyra* and *Bangia* genera. Both agarans and carrageenans are Generally Recognized as Safe (GRAS) compounds widely used in food elaboration.

An *in vitro* study using the microbiota of rats previously adapted to red seaweeds showed that agaran negatively impacted the metabolic activity of the microbiota, reducing its fermentative capacity and SCFA production (83). For these reasons, most of the studies subsequently carried out used oligosaccharides obtained from agarose (AO) or carrageenans (CO) through enzymatic hydrolysis. Han et al. (84) used pig fecal microbiota to ferment AO (neoagarotetraose and neoagarohexaose) and κ-CO for 24 h. Compared with control medium, AO fermentation affected 24 bacterial taxa, increasing the abundances of *Dysgonomonas, Anaerofilum, Enterococcus*, and the butyrate-producing bacteria *Roseburia, Faecalibacterium*, and *Coprococcus*. Accordingly, the concentration of butyrate, valerate, isovalerate, and isobutyrate increased. Regarding κ-CO, 50 bacterial taxa were changed by their fermentation, with increased abundances of *Bacteroides, Enterococcus, Peptococcus, Vellionella, Coprococcus*, and *Roseburia*, and enhanced levels of butyrate. Interestingly, both AO and κ-OC also increased the abundance of *Desulfovibrio*, suggesting that these bacteria could use the sulfate moieties of the algal OS and increase H_2_S concentrations; however, this parameter was not determined. Zhang et al. (88) observed that neoagarotetraose alleviates intense exercise-induced fatigue in mice in association with the modulation of the GM composition and function through increases in Ruminococcaceae, *Roseburia*, and SCFA production. Hu et al. (85) reported an increase of *Bifidobacterium* and *Lactobacillus* species with AO *in vitro* while their administration in mice also increased these taxa, like FOS, and decreased *Bacteroides* and *Enterococcus*. Using AO from *Gellidium* and *Gracillaria* algae cultured *in vitro* with human fecal microbiota, Ramnani et al. (60) did not observed any effect (by FISH) on *Bifidobacterium, Lactobacillus, Bacteroides*, and the butyrate-producing bacteria *E. rectale* and *C. histolyticum*. However, increased acetate and propionate were detected in the culture medium, without changes in butyrate. κ-CO and AO were also degraded by fecal strains of *Bacteroides uniformis* and *Bacteroides xylanisolvens* previously isolated from Chinese individuals and expressing agarase and carrageenase, respectively (86). These results confirm therefore that these enzymes can be expressed in bacteria belonging to the human GM. Finally, Sun et al. (87) reported that the fermentation of κ-OS promoted the growth of *Prevotella* while inhibiting *Bacteroides* and *Parabacteroides*. The fermentation profile of these OS varied according their degree of polymerization, the larger improving SCFA production and the growth of *Bifidobacterium* and *Lactobacillus* while the smaller reduced SCFA production and greatly enhanced the Prevotellaceae abundance. Unfortunately, the culture supernatant of the smaller OS induced higher inflammatory effects on HT29 colonic cells through the increased secretion of IL-1β and TNF-α. Confirming these results, the administration of degraded carrageenan in rats induced TNF secretion and ICAM-1 upregulation in monocytes through NF-kB activation, resulting in the development of colonic inflammation in the animals (89). In another recent study, Shang et al. (90) also described that the administration of κ-, ι-, or λ-carrageenan in mice was found to induce colitis with a similar activity. Notably, all carrageenans decreased the abundance of *Akkermansia muciniphila*, a mucus leaving bacteria known for its anti-inflammatory properties, and negative correlations were observed between the abundance of this microorganism and plasma TNF and colonic histological colonic score in the animals. These results reflect the abundant literature currently available that questions the safety of carrageenan used as food additive.

Globally, these studies suggest that agarans constitute a poorly fermentable substrate for the human GM, but that agarose-oligosaccharides exhibit an interesting prebiotic potential in the pre-clinal models, stimulating butyrate-producing bacteria, bifidobacteria and lactobacilli and increasing the formation of SCFAs including butyrate. Carrageenans also increased SCFA formation but less studies assessing their prebiotic potential are available, probably due to the current concern about their safety. Increases of *Desulfovibrio* were reported in two of these studies. No human studies were performed with agarose-OS to confirm the results of the pre-clinical evaluations. Results from agaran and carrageenan studies are summarized in Table 4.

#### Ulvan

Ulvan is a structural PS from green seaweed of the Ulva genus that exhibits a highly variable and complex structure (Table 2). It is formed of sulfated rhamnose, glucuronic acid and its C5-epimer, iduronic acid, and a minor fraction of xylose and glucose (94). Three main repetitive structures have been observed consisting of units of α-L-rhamnose-3-sulfate-1,4-β-D-glucuronic acid (ulvanobiouronic acid A), α-L-rhamnose-3-sulfate-1,4-α-D-iduronic (ulvanobiouronic acid B), and α-L-rhamnose-3-sulfate-1,4-β-D-xylose. The sulfate groups bound to the ulvan backbone constitute between 12 and 15% of the algal dry matter (69). As other sulfated PS from algae, ulvan has been shown to exert several health-promoting properties including anti-viral, anti-oxidant, anti-coagulant, anti-hyperlipidemic, immunostimulating and anti-proliferative, many of them attributed to its high degree of sulfation and rhamnose content (95). However, few studies have evaluated its ability to modulate GM and prebiotic potential.

One of the first study with ulvan was carried out by Bobin-Dubigeon et al. (91). These authors reported that, after 24 h of incubation *in vitro* with human fecal microbiota, only 25.9% and 50.7% of ulvan and Ulva insoluble fibers, respectively, were degraded, suggesting that ulvan is poorly fermented by colonic bacteria. However, the ulvan constitutive sugars, rhamnose and glucuronate, as well as the ulvanobiouronate repetitive units were highly fermented. To elucidate whether the poor fermentation was due the high content of sulfate and uronic acids, ulvan was desulfated and/or the carboxylic groups of its uronic acid moieties were reduced. These modifications did not affect the fermentation behavior, suggesting that these ionic groups are not implicated in the resistance of ulvan to colonic bacterial fermentation. This is probably due to the type of glycosidic linkages and sequences of sugars forming its structure. Another *in vitro* study with human fecal microbiota confirmed the bad fermentation of ulvan, even after the microbiota was adapted to this substrate (92). At this time, only 8.9% of ulvan organic matter were recovered as SCFAs while the Ulva insoluble fiber (i.e., cellulose, xyloglucan and glucuronan, representing around 13% of the DW) was more fermentable (~50% recovered as SCFAs), confirming the results of Bobin-Dubigeon et *al*. Importantly, around 40% of the ulvan sulfate was dissimilated to sulfide by SRB, increasing therefore, the concentration of H_2_S. In opposition with the previous study, the SCFA production from the desulfated fraction was slightly higher than that of the sulfated, suggesting that the presence of sulfate residues contributes for a small part to its resistance to bacterial degradation. On the other hand, ulvan has been also used as unique source of carbon in pure culture of 17 strains from different species of *Lactobacillus, Bifidobacterium, Bacteroides, Enterococcus, Weissella*, and 7 pathogens, all from commercial culture collections (93). Results showed that only 7 strains belonging to the species of *L. plantarum, B. breve, B. fragilis, B. vulgatus, B. ovatus, B. thetaiotaomicron*, and *B. uniformis* could multiplicate in presence of ulvan, with a moderate growth rate (ΔDO_580nm_: 0.2–0.7) and pH reduction (0.2–0.5). The authors also used human fecal microbiota to evaluate ulvan fermentation for 12 and 24 h. Increased abundances of *Lactobacillus, Bifidobacterium* and *Bacteroides* were observed at 12 h and only of *Lactobacillus* at 24 h, with a moderate production of lactate and acetate after 12 h and 24 h. No animal studies evaluating the impact of ulvan on GM were available.

In summary, the number of studies evaluating the effect of ulvan on the microbiota is low and their results indicate that there is little or no fermentation of this PS by the human GM. Though ulvan lyases belonging to the PL24 or PL25 and PL28 families in the CAZyme database have been involved in ulvan degradation, these enzymes, described in marine bacteria, are probably absent in the bacteria from the resident GM in humans. In addition, the transformation of part of its sulfate residues to H_2_S by SRBs in the colonic lumen could have a negative impact on the digestive ecosystem and host's health. Based on these results, it can be concluded that ulvan probably cannot be considered as prebiotic. Results from ulvan studies are summarized in Table 4.

## Conclusions

**Figure 1:**
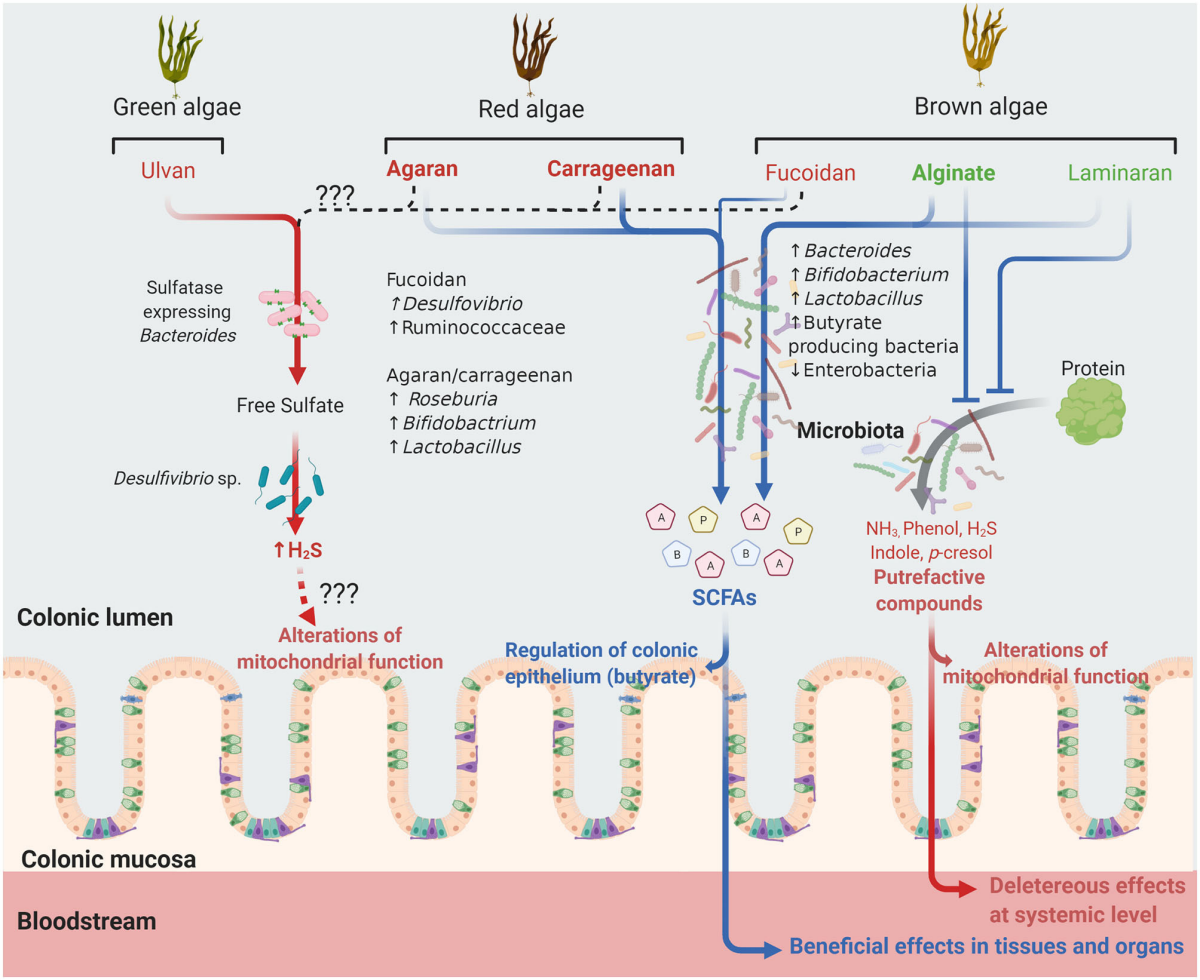
Future of undigestible algal polysaccharides in the colonic lumen. Polysaccharides (PS) from different algal families are shown. Those in green are non-sulfated PS, those in red sulfated PS and those in bold character have gelling properties. Laminaran and alginate increase the abundance of the bacterial taxa indicated, generating short chain fatty acids (SCFA, blue arrow) including acetate (A), propionate (P) and butyrate (B). These PS also interfere with metabolic activities and/or bacterial populations involved in protein degradation and fermentation, reducing the subsequent formation of putrefactive compounds potentially deleterious at local or systemic levels. Carrageenan and agaran, mainly as oligosaccharides, can also be fermented, generating SCFA while fucoidan and ulvan are apparently less fermented. In the case of ulvan, *in vitro* studies indicate that its metabolism by the microbiota, more particularly bacteria from the *Desulfovibrio* sp. and eventually sulfatase expressing *Bacteroides* sp., result in the formation of hydrogen sulfide (H_2_S) (red arrow) that, when produced in excess, can inhibit the colonocyte mitochondrial function. It is unknown whether fucoidan might also result in H_2_S production (dashed black arrow).

The global market for algae products is expected to reach $ 6.4 billion in 2026, owing to the increasing applications of micro and macroalgae not only in food, cosmetics, and pharmaceuticals, but also in the areas of agro-industry, bioremediation, biodiesel, and biodegradable plastics. To respond to the growing requirements of these sectors, more environmental-friendly technologies have been developed such as supercritical fluid, microwave-assisted, enzyme-assisted, and pressurized-liquid extraction methods that allow to obtain with a higher efficiency and lower cost algal bioactive compounds of interest including polyphenols, long-chain polyunsaturated fatty acids, pigments, enzymes, dietary fibers and proteins. In the areas of health and nutrition, there is a huge interest in generating scientific evidence supporting the health promoting properties of these algae-derived ingredients and, therefore, the expansion of this market. Our review falls within this context, seeking to critically determine the current level of evidence supporting the existence of prebiotic properties for seaweed PS. Our results, summarized in Figure 1, suggest that non-sulfated PS are more fermentable by the GM, promoting SCFAs formation, and *Bacteroides, Bifidobacterium, Lactobacillus* and butyrate-producing bacteria growth, whereas attenuating the production of harmful putrefactive compounds. Sulfated PS are less fermented in their native form but their use as oligosaccharides improves their fermentability and prebiotic properties. The impact of fucoidan on the GM is highly variable and few studies are available with ulvan. Notably, part of the ulvan sulfate residues is transformed to H_2_S while there is no data available for fucoidan. Although several studies suggest that algal PS improve metabolic parameters in animal models of metabolic diseases, it is unclear whether these effects are related to microbiota regulation. According to the current evidence, it can be concluded that alginate, laminaran and agaran-OS have interesting prebiotic potential while that of fucoidan and ulvan is questionable. However, the development of clinical trials in healthy volunteers is a *sine qua non* condition to confirm their eventual prebiotic properties and the absence of adverse effects.

## Author Contributions

MG was responsible for the conception and organization of the manuscript and wrote the sections Some Concerns About the Use of Algal PS as Prebiotics and Conclusions. SR, AB, and FM contributed to the sections Introduction, Algal polysaccharides, Degradation of dietary PS in the colon, The concept of prebiotics, and The evaluation of PS prebiotic activity. KR and NG to the section Non-sulfated PS and CC and GL to the section Sulfated PS. All authors contributed to manuscript revision and read and approved the submitted version.

## Conflict of Interest

The authors declare that the research was conducted in the absence of any commercial or financial relationships that could be construed as a potential conflict of interest.
